# Need for operational simplicity and timely disbursal of benefits—a qualitative exploration of the implementation of a direct benefit transfer scheme for persons with tuberculosis in India

**DOI:** 10.1186/s40249-024-01206-3

**Published:** 2024-05-23

**Authors:** Malu Mohan, Jeromie W. V. Thangaraj, Sumit Pandey, G. Sri Lakshmi Priya, Sivavallinathan Arunachalam, Rahul Sharma, Hemant Deepak Shewade, B. Aishwarya, K. Afeeq, Afsana Khatoon, B. Gokulvijay, Gude Sireesha, Kavita Chandra, S. Nandhakumar, Prince Samuel, C. Nanditha Viswanathan, Devika Shanmugasundaram, Raghuram Rao, Manoj V. Murhekar, Kathiresan Jeyashree

**Affiliations:** 1 Indian Council of Medical Research-National Institute of Epidemiology, R-127, TNHB, Ayapakkam, Chennai, Tamil Nadu 600077 India; 2 TB Support Network, WHO Country Office for India, New Delhi, India; 3 Central Tuberculosis Division, Government of India, New Delhi, India

**Keywords:** National Tuberculosis Elimination Programme, Nutritional status, Direct benefit transfer, *Ni-kshay Poshan Yojana*, Implementation, Cash transfer scheme, Grounded theory, India

## Abstract

**Background:**

*Ni-kshay Poshan Yojana* (NPY) is a direct benefit transfer scheme of the Government of India introduced in 2018 to support the additional nutritional requirements of persons with TB (PwTB). Our recent nationwide evaluation of implementation and utilization of NPY using programmatic data of PwTB from nine randomly selected Indian states, reported a 70% coverage and high median delay in benefit credit. We undertook a qualitative study between January and July 2023, to understand the detailed implementation process of NPY and explore the enablers and barriers to effective implementation and utilization of the NPY scheme.

**Methods:**

We followed a grounded theory approach to inductively develop theoretical explanations for social phenomena through data generated from multiple sources. We conducted 36 in-depth interviews of national, district and field-level staff of the National Tuberculosis Elimination Programme (NTEP) and NPY beneficiaries from 30 districts across nine states of India, selected using theoretical sampling. An analytical framework developed through inductive coding of a set of six interviews, guided the coding of the subsequent interviews. Categories and themes emerged through constant comparison and the data collection continued until theoretical saturation.

**Results:**

Stakeholders perceived NPY as a beneficial initiative. Strong political commitment from the state administration, mainstreaming of NTEP work with the district public healthcare delivery system, availability of good geographic and internet connectivity and state-specific grievance redressal mechanisms and innovations were identified as enablers of implementation. However, the complex, multi-level benefit approval process, difficulties in accessing banking services, perceived inadequacy of benefits and overworked human resources in the NTEP were identified as barriers to implementation and utilization.

**Conclusion:**

The optimal utilization of NPY is enabled by strong political commitment and challenged by its lengthy implementation process and delayed disbursal of benefits. We recommend greater operational simplicity in NPY implementation, integrating NTEP activities with the public health system to reduce the burden on the program staff, and revising the benefit amount more equitably.

**Supplementary Information:**

The online version contains supplementary material available at 10.1186/s40249-024-01206-3.

## Background

Poverty, food insecurity, under nutrition, overcrowding and other forms of deprivation are proximate risk factors of tuberculosis (TB), which are also implicated in reduced access to TB care, high TB incidence and mortality [1–3]. Poor nutritional status has been identified as a risk factor for TB incidence and death [4, 5]. A cluster randomised trial conducted in the Indian State of Jharkhand reported that nutritional intervention was associated with substantial (39–48%) reduction in tuberculosis incidence in the households of microbiologically confirmed pulmonary TB cases during 2 years of follow-up [6]. Additionally, nutritional support of persons with TB (PwTB) with a high prevalence of severe under nutrition was associated with significantly reduced TB mortality, better adherence, treatment success, and weight gain [7].

Over the past four decades, global initiatives for the prevention and control of TB have predominantly been within a germ-centric and treatment-based biomedical paradigm, focused largely on the systemic approaches of case detection and care, and barely addressing primary determinants of health [8]. Since 2000, the international development goals, policy initiatives and national health programs for TB have adapted to the emerging discourse around social determinants of health [9]. The End TB strategy also acknowledges the need to enhance access through universal health coverage policy, poverty alleviation, and action on other health determinants in addition to biomedical and public health strategies [10].

Social protection interventions, including poverty eradication schemes for PwTB and TB-affected households, are increasingly being incorporated into national TB program strategies. In low- and middle-income countries, nutritional support in kind constitutes the highest proportion (30%) of schemes for TB-affected households, followed by conditional cash transfer [11]. Cash transfer schemes constitute a TB-specific approach to cover TB-related costs in households affected with TB and target treatment success [12–14].

*Ni-kshay Poshan Yojana* (NPY), (*Ni-kshay*- free of TB, *Poshan*- nutrition, *Yojana*- scheme) was launched as a nationwide direct benefit transfer (DBT) program by the Government of India on 1 April 2018 [15]. Under this scheme, all PwTB, notified under the *Ni-kshay* portal (an electronic web-based notification and information management system) receive financial assistance of INR 500 (USD 6) per month to PwTB for nutritional support directly transferred to their account for the duration of anti-TB treatment [15–17]. NPY completed five years of its implementation in 2023, and, the Central TB division (CTD), Ministry of Health and Family Welfare, India, requested us to conduct a nationwide comprehensive evaluation of its implementation and utilization. Such an evaluation would allow the analysis of systematically collected data on scheme implementation, within an implementation science framework, to identify the enablers and barriers, find appropriate solutions to overcome the barriers and promote sustainability [18]. Our quantitative analysis of programmatic data of PwTB in nine randomly selected Indian states reported a 70% coverage and longer median delay in benefit credit [19]. For an in-depth understanding of these results, we conducted a qualitative exploration of the 'how' and 'why' of program implementation, to develop a comprehensive understanding of the actual implementation process, the enablers, and barriers to its optimal implementation.

## Methods

### Reflexivity statement

The research team had their basic training in modern medicine or allied health professions, with postgraduate training in public health. They were primarily trained in post-positivist research paradigms, but were adequately exposed to constructivist, transformative and pragmatic paradigms through training, work experience, or both. The team was diverse in gender composition, linguistic and cultural origins and the nature of the research experience, which aided in more nuanced transpersonal reflexivity.

### Study setting

India is administratively divided into states or union territories, and each state or union territory is divided into districts. Under the National TB Elimination Program (NTEP), the district TB centres led by the district TB officer (DTO) monitor the program implementation through a network of tuberculosis units (TUs) and peripheral health institutions. The state TB cell, led by a state TB officer (STO), governs all district TB centres.

NPY implementation broadly involves registering the notified patient for the benefit using their bank account. The list of PwTB due for the benefit every month is approved by the DTO and forwarded to the Public Finance Management System (PFMS) for validation of bank accounts and credit of benefit to the patient's bank account. The benefit for the first two months of treatment is credited together as the first instalment of INR 1000 (USD 12) upon notification of the patient, and the subsequent instalments of INR 500 (USD 6) are credited monthly for the rest of the treatment period. This process is repeated every month until the treatment outcome is declared for the patient [15–17].

### Study design

**Fig. 1:**
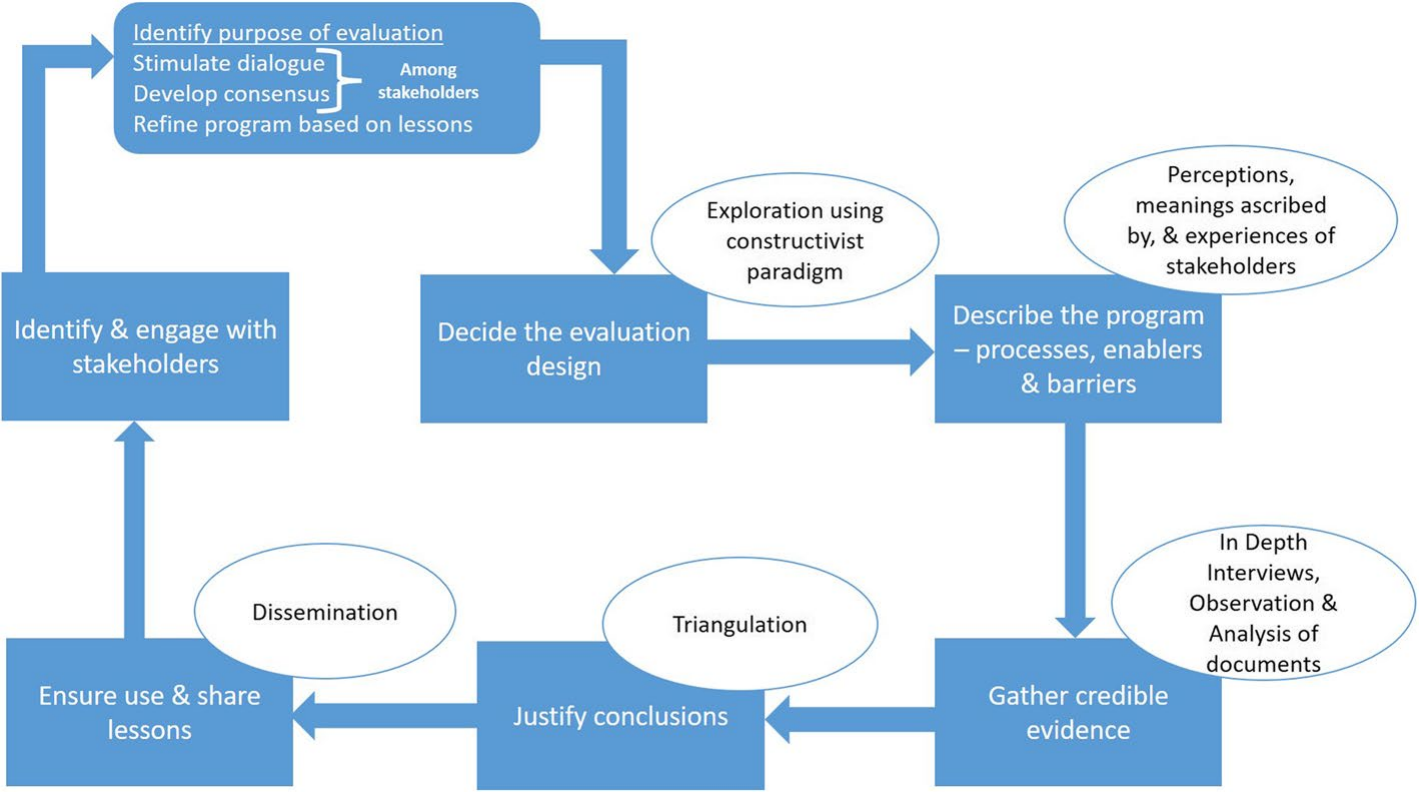
Conceptual framework of the exploration of *Ni-kshay Poshan Yojana* in India, 2023

The enablers and challenges encountered in implementing NPY would be best understood within a constructivist paradigm, acknowledging the social situatedness and contextual relativism of the knowledge [20]. We conceptualized program implementation as a complex, multidimensional phenomenon deeply influenced by the diverse contexts and the stakeholders' perceptions from various levels of program implementation and the beneficiaries (Fig. 1). We relied on the perceptions, meanings ascribed by and the experiences of the stakeholders involved in the scheme to finalize the purpose and design of the exploration. A detailed description of the implementation process in real settings was concluded as a necessary prerequisite in the meticulous documentation of enablers and barriers to implementation and utilization. The exploration followed an iterative and cyclical process of data generation, triangulation and dissemination through constant stakeholder engagement.

A grounded theory approach was used to systematically construct the detailed processes, and to develop a comprehensive explanation of the challenges in implementation and utilization by identifying the enablers and barriers encountered [21]. Grounded theory is a qualitative research approach which aims to develop theoretical explanations for social phenomena (how and why) through data generated from multiple sources by way of observations and interviews, rather than testing hypotheses based on pre-existing theories. In this study, we have followed the steps stipulated in the approach by simultaneously conducting data collection using theoretical sampling, and inductive data analysis where analytical codes and categories are constructed using constant comparison method [21]. The approach is further detailed in the subsequent sub-sections – sampling and data analysis.

Accordingly, we developed preliminary framework of the stipulated process of NPY implementation from the scheme-related documents, which was then expanded through observations and interactions with the stakeholders from the health system at the state, district and field levels. The processes were converted into pictorial representations with increasing detail, referred to as process maps, for easier and effective communication with the stakeholders. Concurrently, the enablers and barriers to implementation and utilization were also explored and the barriers encountered were indicated within the process maps.

### Study participants

We selected the following categories of participants from the nine states sampled for the larger study, the sampling methods of which are described by Jeyashree et al. 2024 [19].

i. Registered beneficiaries of the scheme.
ii. Field health workers/volunteers from the public health system and non-governmental organizations (NGO).
iii. District/state level program officers, TB consultants from the World Health Organization (WHO) involved in NPY, and key officials from the CTD.

### Sampling

We used theoretical sampling in the study, where the developing theoretical categories direct the researchers to pertinent data sources and participants, which will explicate and advance the theoretical categories [21]. We started the data collection by analyzing the key scheme-related documents and interviewing the key officials from the CTD, followed by the concerned program officers from the selected states and districts. Data collection coincided with the data analysis and as categories on implementation process emerged, data was also collected from WHO consultants, field level officials and beneficiaries across the districts. The sampling process acknowledged the contextual differences across study settings in terms of participants, processes, enablers and barriers. For instance, in one of the eastern Indian states, we interviewed the Accredited Social Health Activists (ASHAs) who were known to play a crucial role in NPY implementation. In another north Indian state, we interviewed specific non-governmental agencies and their volunteers to shed further light on the implementation process. As the data collection and analysis continued, the properties of each category/theme were saturated and comprehensive explanations driven by empirical data emerged, at which point further data collection was terminated.

### Data collection

Data collection for the study occurred between January and June 2023. The primary data collection method was face-to-face, in-depth interviews using interview guides developed by the research team in alignment with the objectives and the conceptual framework (Supplementary files 1, 2 & 3). The interviews were conducted by a team of experienced data collectors led by the first authors. The data collection team was fully oriented to the objectives of this study and trained to use the interview guides. The interviews were conducted in the language of choice of the respondents which was mostly English, Tamil, Telugu or Hindi. For data collection in other states, we had employed the help of formal translators to conduct the interviews.

The stakeholders were initially approached through email and or phone calls seeking appointments for interviews. At that stage, some of the stakeholders initially expressed uncertainty/unwillingness and sought further clarifications primarily regarding data confidentiality. Once the relevant clarifications were provided, all the stakeholders agreed to the in-depth interviews. Additional interviews were conducted virtually with the district/state level stakeholders and officers from the CTD if further clarifications were necessary. Some health system stakeholders were acknowledged as key informants, considering their expertise, and were repeatedly interviewed for clarifications and validation throughout the study. The field notes of the data collection team and scheme-related documents and reports were used for data source and methodological triangulation.

After the process of field data collection, a consultative meeting of DTOs from the study districts and the officers from the CTD was organised for theoretical triangulation; to refine and validate the constructs that emerged from the ground. The stakeholders reviewed the process maps and listed the enablers and barriers encountered at each stage of NPY implementation.

### Data analysis

The scheme-related documents and reports were inductively analyzed to identify the preliminary thematic categories. The audio recordings of interviews were transcribed verbatim in English, and a team of three coders worked on the data analysis. Six interviews were inductively coded separately by two coders, and the conceptual differences were sorted through detailed discussions to develop an analytical framework through consensus. Subsequent interviews were coded, guided by the analytical framework. New concepts or categories which emerged during the analysis were added to the existing framework. Categories and themes emerged through constant comparison [21]. Coding, forming categories and themes, and data collection through theoretical sampling aligned until the theoretical categories were saturated and the patterns across the themes had emerged. We have followed the consolidated criteria for reporting qualitative research (COREQ) guidelines (COREQ checklist – supplementary file 4) for reporting this study [22].

## Results

We conducted 36 in-depth interviews with different stakeholders Table 1.

**Table 1:** Details of the in-depth interviews conducted with stakeholders in *Ni-kshay Poshan Yojana* implementation in nine states of India, 2023 (*n* = 36)

Category of participants	Number of interviews	Range of duration of interview in minutes
Central TB division/state/district level TB officers (district tuberculosis officers/ district program coordinators)	17	7–41
Field/tuberculosis unit level health workers/volunteers (including members from non-governmental organizations)	9	8–24
Beneficiaries	10	10–15

The major themes which emerged from the analysis were i) implementation process of NPY, ii) enablers and iii) barriers to implementation and utilization (Table 2).

**Table 2:** Themes and sub-themes on the exploration of process of implementation, enablers, barriers to implementation and utilization of *Ni-kshay Poshan Yojana* in India, 2023

Themes	Sub-themes
Implementation process	a) Collection and validation of bank account details of beneficiaries b) Benefits' approval and credit of payments
Enablers of implementation and utilization of NPY	a) Political and administrative commitment to implementation b) Public transportation facilities c) Sub-national initiatives
Barriers to implementation of NPY	a) Opening bank accounts b) Physical access to banks and health facilities c) Technical issues related to computer access, internet connectivity and *Ni-kshay* portal d) Hesitation and unawareness of the beneficiaries e) Human resource constraints f) Approval process of benefits g) Other factors
Barriers to utilization of the benefits	a) Perceived inadequacy of the benefit amount b) Delay and uncertainty in the credit of payments c) Banking related issues d) Transportation facilities and road connectivity

### Theme 1: implementation process

**Fig. 2:**
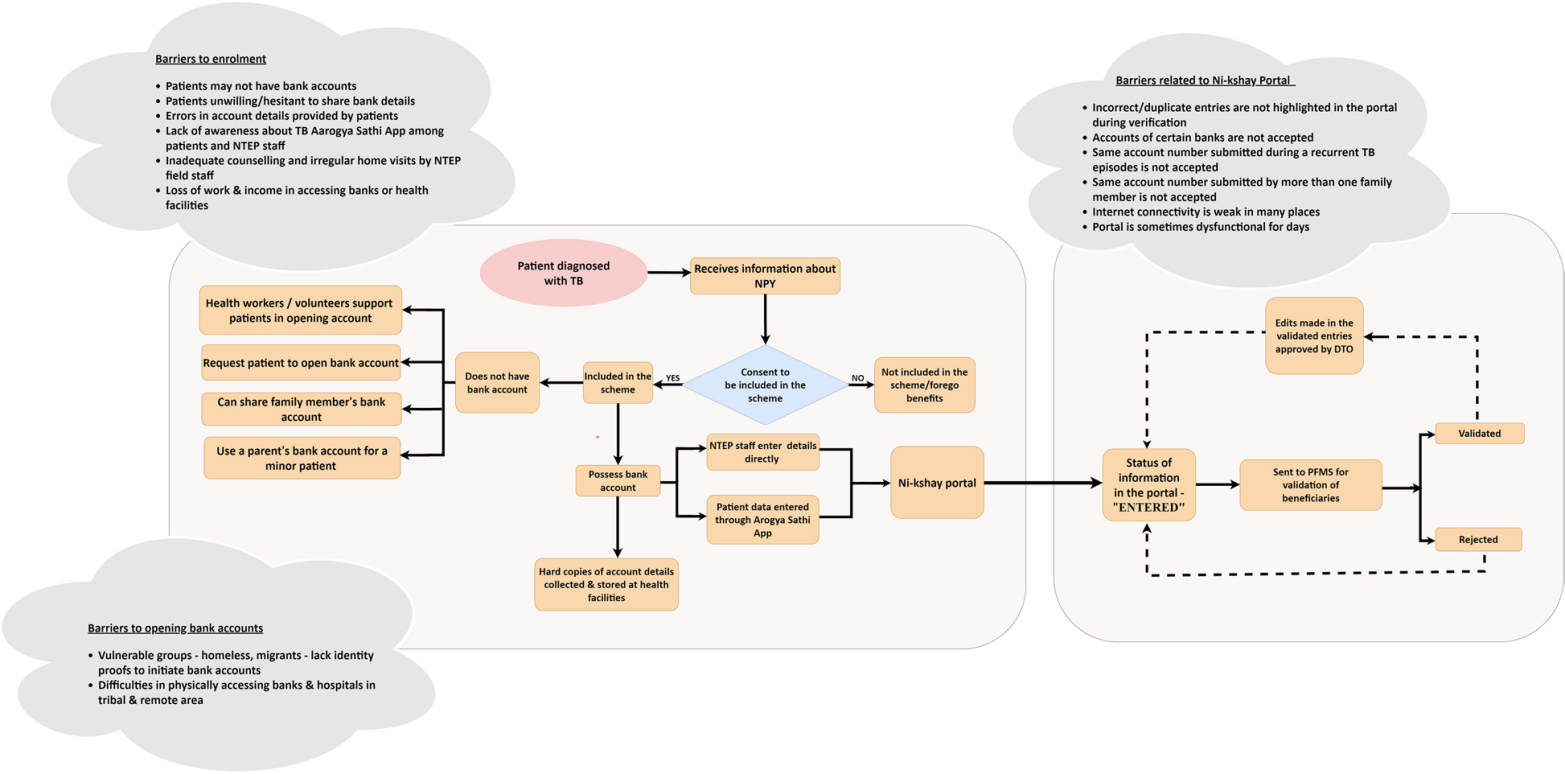
Barriers in the collection and validation of bank account details of beneficiaries—implementation of *Ni-kshay Poshan Yojana* in India, 2023

**Fig. 3:**
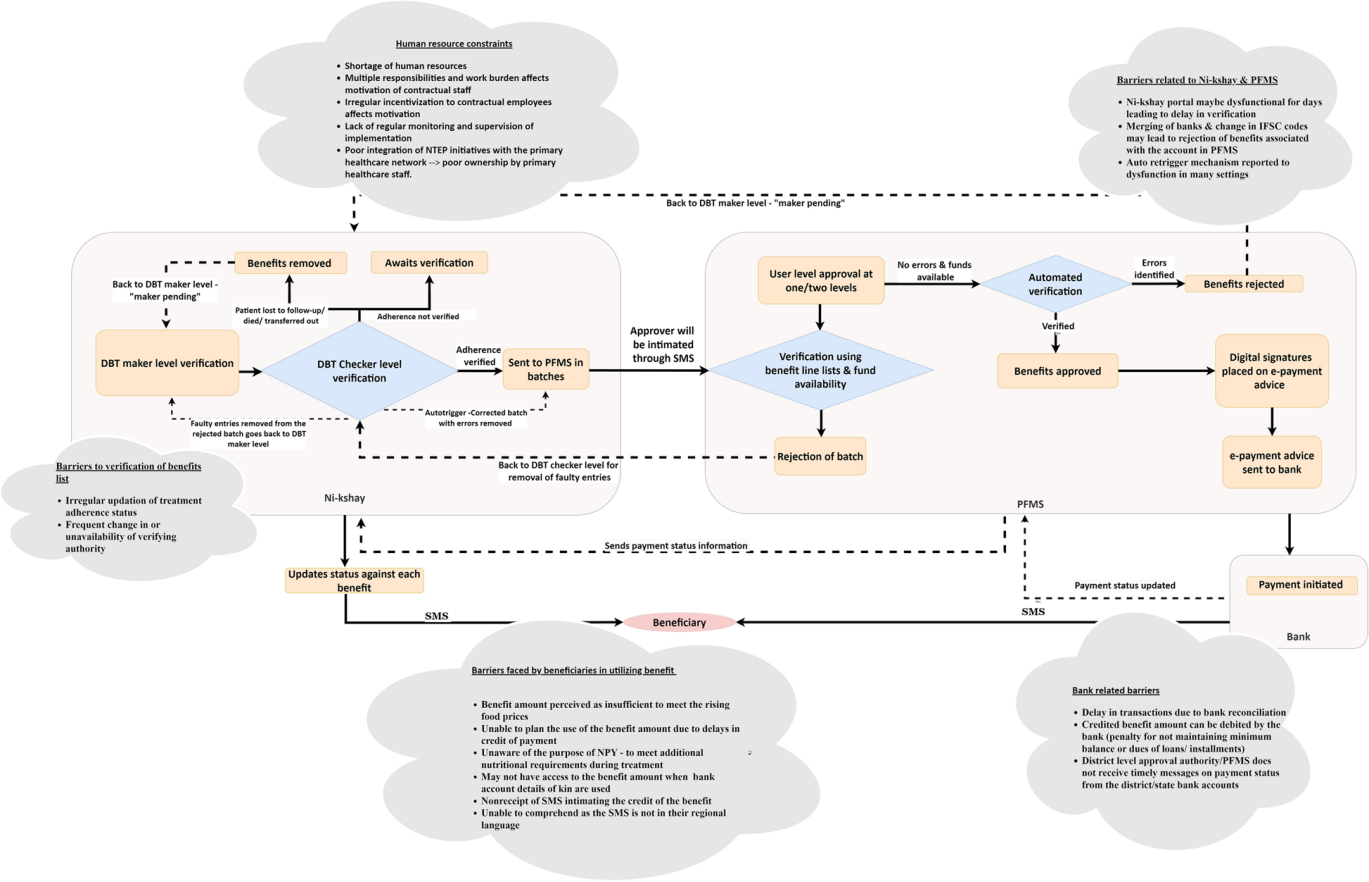
Barriers in benefits' approval and credit of payments- implementation of the Direct Benefit Transfer (DBT) scheme—*Ni-Kshay Poshan Yojana* in India, 2023

The implementation had two major processes namely, the collection and validation of bank account details of beneficiaries (Fig. 2) and the approval of benefits and credit of payments (Fig. 3).

#### Collection and validation of bank account details of beneficiaries

Every eligible beneficiary under NPY should have a unique bank account into which cash will be directly credited in monthly instalments throughout treatment. The hard copies of the bank account details and relevant identity proofs are brought to the health facilities by potential beneficiaries/family members or collected by the field workers through home visits. The account details are entered into the *Ni-kshay* portal either directly by the field staff or via the TB Aarogya Sathi app [23]. Those beneficiaries who do not have bank accounts are requested to open an account for the purpose and are supported by the field staff in this process if required. They can also use a family member’s account to receive the benefits after declaring the familial kinship of the account holder with the beneficiary.

*“The patient can download the app and just fill in their bank account details and it is linked to Ni-kshay. Even after they feed their bank account details, ultimately this has to be approved by the NTEP staff there. The bank account details have to be collected from the patient by the NTEP staff. So, home visits and verification are an integral part of implementation.”*- National-level NTEP official

The information regarding a potential beneficiary “entered” in *Ni-kshay* is automatically transferred to the PFMS to validate the bank account details online. PFMS is a web-based online software application under the Government of India that tracks the funds released under all welfare schemes, including the direct payments to the beneficiaries and real-time reports of the expenditure involved at all program implementation levels. The beneficiary accounts are either validated or rejected based on the veracity of the account details.

#### Benefits' approval and credit of payments

Once the bank accounts are validated at the PFMS, the approvers at the district level (DTO/ City Health Officer/District Medical Officer/District Collector) are intimated via short message service (SMS), who then approve or reject the payment based on fund availability at the state treasury. Despite differences across states, the approval process mostly happens at the district level. In one of the southern states, this approval takes place in a single step at the state level (instead of at the district level), reducing processing time and avoiding administrative delays.

A list of benefits is generated in the *Ni-kshay* portal, which the program staff verifies at two levels. First, the program staff at the TU level, designated as “DBT makers” in the scheme, verifies the list of validated beneficiaries eligible for each instalment of benefits generated in the *Ni-kshay* portal. Next, the DTO designated the "DBT checker" verifies the benefits after which the benefits are grouped into batches in *Ni-kshay* and sent to PFMS for verification and approval.

*"At the TU level there would be STS, lab supervisor, health volunteer, whoever is in charge, given the responsibility of a maker, to verify the list in Ni-kshay. And thereafter, at the district level is the Checker login, it can be DTO, it can be the accountant, as authorized for that purpose."*- National level NTEP official

*"So, the maker will approve it and send for approval to DTO, who is a checker. The checker will approve it, and it will go to the PFMS. Now, depending on the state, it may change, but in the PFMS, someone has to give financial approval."*- National-level NTEP official

Once the PFMS approves the release of funds, the payments are credited to the beneficiaries. On receipt of e-payment advice, the bank initiates payment, and the payment status is updated in the PFMS. Once the benefit is credited to their account, the beneficiaries receive an SMS from *Ni-kshay* and the bank.

If errors are identified in even a single benefit sent from the district, the whole batch is rejected in the PFMS. Then, the DBT checkers identify and rectify the erroneous entries in the *Ni-kshay* portal. The revised batch of error-free benefits is automatically sent to PFMS for approval, a process called the *auto retrigger* mechanism.

### Theme 2: enablers of implementation and utilization of NPY

NPY was perceived as a very helpful initiative by the providers and beneficiaries. It was considered especially beneficial for the poor and socially marginalized sections, including the elderly, tribes, and those residing in remote and difficult terrains.

*"This DBT scheme (NPY) is very good. The government provides nutrition to the patients because most of them are malnourished, and they are affected by TB mainly because they are malnourished. More and more patients are joining for treatment."- *District-level NTEP official.

*“The scheme was very helpful. Because of the scheme I was able to take fruits and vegetables and care for my health while I was taking treatment. I got the full amount after treatment. There was some delay, but I got it fully.”*- NPY beneficiary who completed the treatment.

Many providers observed that the scheme enhanced the notification of TB cases and improved the tracking of cases. They also suggested that the direct transfer of money to the beneficiary accounts enhanced transparency and reduced the chances of corruption.

#### Political and administrative commitment

In one of the southern states, the federal government and the district administrations displayed a relatively higher political and administrative commitment to implementing the scheme. Monitoring and supervision of the scheme implementation and extent of utilization were conducted through regular review meetings by the state's Chief Secretary to the federal government. Regular review meetings were also followed at district and TU levels, enabling the officials to effectively implement the scheme, redress the grievances on time, and enable the state to work closely with the private sector facilities.

*"When we started the implementation, initially we had the Chief Secretary reviewing the program every month. Also, we had district and state level officials reviewing this program almost on a 15 days basis. So, there was a lot of political and administrative commitment given and because of that we felt that the processes were made more efficient."*- District WHO Consultant

The recent initiative of the central government to upgrade the health sub-centres, the basic units of India's primary health care network, to Health & Wellness Centres (H&WCs) had greatly reduced the workload of TB health workers.

"*Health and Wellness Centres have now started, and so now we have more people helping us with the field work at the village level."*- District NTEP staff

Patient-Provider Support Agency (PPSA) is a "model under which a state/district TB unit contracts a third-party agency/NGO to engage private-sector doctors and provide end-to-end services". PPSA is a key enabler in supporting the collection of bank account details of potential beneficiaries in private-sector health facilities and follow up with the credit of the benefits. Since the introduction of this initiative in 2021 there has been an increase in PwTB using the private sector receiving the NPY benefits.

*"PPSAs take care of the private patients, right from diagnosis to outcome, including all public health activities. So, because of them, from mid-2021, the gap in reaching the private sector patients has decreased in some parts of the state.*"- District NTEP staff

#### Public transportation facilities

In some study settings, effective public transportation facilities made it conducive for PwTB to travel to health facilities to enrol in the scheme and to banks to open accounts or withdraw the benefit amount. Transportation and road connectivity were also major enablers for the health workers to contribute to the scheme implementation through regular home visits.

#### Sub-national initiatives

One of the north-eastern states initiated a digital application to track real-time maternal and child health (MCH) data from all the health facilities. The application also tracks PwTB, enabling the program staff to promptly reach out to those seeking treatment in private health facilities, thus ensuring their enrolment in NPY benefits without delay.

"*This MCH app is like a step in between. All the patient information is already available from it. So, we will get the patient's details from the app and enter into Ni-kshay."*- District NTEP staff

In one of the north Indian states, a grievance redressal mechanism was initiated by the state (sub-national) government to address the beneficiaries' concerns regarding government schemes, including NPY. The beneficiaries can access grievance redressal through a toll-free phone number. The stakeholders reported this mechanism as more effective than the routine grievance redressal mechanism instituted through *Ni-kshay*.

In one of the northern states, a non-governmental organization collaborated with the district government hospital to test and treat homeless people in shelter homes. If the PwTB had at least one identity proof, the organization assisted them in opening bank accounts to access NPY benefits. Since they did not have access to the amenities required for cooking, the organization provided them with food throughout treatment. In another study setting from the northeast, the district head led a campaign to promote Aadhar Card (identity card with a unique identity number that can be obtained voluntarily by all residents of India) registration. In this state, where a large migrant population and people from tribal communities typically struggle or hesitate to register for identification documents, this proved to be beneficial. As identification documents are required to open bank accounts, this drive also helped NPY implementation.

### Theme 3: barriers to implementation of NPY

#### Opening bank accounts

PwTB who do not possess individual bank accounts cannot receive NPY benefits. The same account cannot be used again if a patient suffers more than one TB episode. Family members with more than one PwTB cannot use the same account even if the PwTB is a minor or elderly who can have difficulty in accessing banking facilities. Thus, many beneficiaries are required to open new bank accounts to be enrolled in NPY. In several ethnic groups, the concept of "banking" itself is alien, which makes it difficult for health workers to convince them regarding the importance of opening bank accounts for NPY.

*"I feel bank account opening is the major problem. Patients do not have bank accounts most of the time and they need to spend a lot of time, money and effort to open the accounts. We are helping them start bank accounts, but some of them don't even have ration cards and forget Aadhar cards. So, then we have to start there."*- Field level NTEP staff

Vulnerable populations like people experiencing homelessness or migrants may not have documents that prove their identity proof to initiate bank accounts. Thus, the neediest people, such as the homeless, destitute and the cognitively challenged, were not included in the scheme due to difficulties in opening bank accounts.

"*There are some people who are homeless. They stay on the railway platforms or at the bus stand, or under the tree or somewhere under the flyover bridges. These people don't have any documents with them, and opening a bank account will be very, very difficult.*"- District WHO Consultant

*"But if you see, these are the most deserving people who need this money, but somehow, we are not able to give them the entire money."*- Field level NTEP staff

*“The people that we are working on are not suitable for the scheme because they don’t have any formal documents proving their identity. The scheme may be useful for many people, but not for the people that we are working with… I don’t think it is of much help. We would like the governments to place more attention to the PwTB who are on the sidelines and who are too marginalized to fulfil the necessary conditions to receive such support.”*- Representative, NGO working on homeless and marginalized sections

There were also instances where the PFMS does not accept the accounts of local banks like the Grameen (Village) or the Cooperative Bank, which also led to rejections of bank accounts requiring beneficiaries to open new accounts in other banks.

#### Physical access to banks and health facilities

In some tribal and remote areas, there were challenges physically accessing the banks and health facilities due to poor road connectivity and lack of transportation facilities. This led to missing work and loss of income, which was also a barrier to opening bank accounts.

*"Travel is an issue here. As you saw, the roads are bad, winding. Transportation is a problem. We struggle to reach them (beneficiaries). They struggle to reach us also. This is even bad when it rains. Then it is beyond any discussion".*- Field level NTEP staff

#### Technical issues related to computer access, internet connectivity and Ni-kshay

Internet connectivity and computer access are prerequisites to access the *Ni-kshay* portal, and several of the TUs under study did not have working equipment or uninterrupted internet services.

"*There is no computer here. One tab has been given to each of us, but they are having issues now. These were given about 2–3 years back. They cannot be replaced…no repair can be done. We are using the mobile phone itself."*- Field-level NTEP staff

Sometimes, *Ni-kshay* portal was not functional for several days together, snowballing into accumulated unpaid benefits and major delays. Obtaining technical support to address such glitches was reported to be difficult. The program staff struggled to navigate the digital platforms, caused partly by their inadequate technical skills, highlighting the need for better training modalities to support them in operating these platforms.

*"Sometimes Ni-kshay doesn't open. It doesn't bring up the page soon. The Internet is weak here, that is also an issue, especially when we go into the villages. In most places, there is no internet connectivity. So, if I have to check someone's payment status, I will have to call the STS. Then make them check. These issues keep happening in the village side."*- Field-level NTEP staff

#### Beneficiary-related challenges

PwTB from the private sector facilities hesitate or are unwilling to share their details with the health workers leading to a delay in enrolment and subsequent processes. Many PwTB did not have the required information about NPY and its processes, which prevented them from enrolling in the scheme and sharing their bank details. The district-level stakeholders attributed this to the deficiencies in the program staffs’ engagement with the patient, counselling and irregular home visits.

"*Problems from the patient's side are usually related to their unawareness of the scheme. They are not properly informed that the money is being provided for nutritional purposes. They don't have proper bank accounts; they are hesitant to share the details, and they don't bring the documents on time, which results in delay in validation of the account.*"- District-level NTEP staff

#### Human resource constraints

The shortage of motivated human resources was a critical barrier. The existing program staff catered to much larger populations than originally stipulated and felt overburdened with the multiple responsibilities in the program leaving no time for engagement about NPY. Many critically observed that no additional human resources were recruited to implement NPY.

"*The guideline was that there should be one senior treatment supervisor (STS) per 1.5 lakh population. However, if I give the example of this district, there is 1 STS per 3 lakh population, and there is a human resources requirement, there are vacant posts. Until human resource (HR) is not improved, the program can't be improved. Because in NTEP, new schemes have started, like DBT. But there are no human resources for that. If the STS keep working on other TB programs, they cannot do their routine work. So, in order to improve this program, the government should employ regular staff in this."*- Field-level NTEP staff

*"The issue is severe HR shortage. Plus, they are not at all motivated. If you look at my district, I am the maker, checker and approver most of the time. Many of the program staff are on a contractual basis. They are overworked. See madam, NPY is a separate scheme but there is no separate HR for it. It is done by the same program staff who have to do case finding, investigations, treatment, monitoring adherence and documentation. So, we cannot put pressure on them beyond a level."*- District-level NTEP staff

The field workers observed that the health system currently conceived TB-related work as almost an exclusive responsibility of the NTEP staff despite efforts to mainstream the program work with public health work at primary health care level. Digital documentation in *Ni-kshay* was an added responsibility for the program staff, and the lack of adequate supportive supervision and technical support when required added to their workload.

*"The staff is struggling with all these schemes. We have to document everything, right? In fact, I think TB should be the problem of all the health staff, not just us (NTEP staff). Getting the cooperation from them (the health staff from the primary health care network) is so difficult. They are also busy. We are also busy."*- Field-level NTEP staff

*“…Sometimes we have some issues with the Ni-kshay updates. If we have an issue, there is no one to turn to immediately to ask for advice or help. Already we are struggling … Sometimes, the tablets or even the desktop at the TU gets repaired and it takes a long time to get them to work. So then we use our own phones for documentation, which sometimes don’t work properly…”*- Accredited Social Health Activist (ASHA worker)

#### Lengthy approval process

The stakeholders perceived the benefit approval as complex and laborious, both process-intensive and labour-intensive. Currently, this process is undertaken at the district level in most states. The approval process could be delayed due to various reasons, which include the unavailability of concerned authorities in the posts due to leaves/transfers, frequent changes in approving authority, editing the details after validation (which requires additional approval), merging of banks and changes in the Indian Financial System Codes (unique identifiers of Indian banks) resulting in non-validation of bank accounts, bureaucratic delays in file transfers, lack of adequate funds or competing financial priorities such as salaries.

*"Delays are there. But that I think is because the money is processed at the district level instead of lower levels. District approval takes so much time. If the concerned officers are busy or on leave, then it takes so much more time."*- District-level NTEP staff

"*There are many red tapes in the process. This file must go to the district health officer and then to the district collector. Without the district collector's permission, we can't do anything."*- District-level NTEP staff

Inter-state variations in the availability of funds for NPY, frequent changes in fiscal policies about NPY by the state or central governments, including the changes in banks, affiliated PFMS agencies and approving authorities, non-payment of benefits to beneficiaries after six months of treatment (persons with drug-resistant TB and persons with drug-sensitive extra-pulmonary TB), erroneous rejection of beneficiaries were among the other challenges reported by some states. In the northern states, the district officials could not process the NPY benefits during the reconciliation period of banks, which would occur every three months.

### Theme 4: barriers to utilization of the benefits

#### Perceived inadequacy of the benefit amount

The stakeholders also highlighted that while the current benefit amount was helpful for few beneficiaries, it was barely adequate for several others. Thus, the utility of the benefit also depended on the socio-economic circumstances of the beneficiaries and households.

*"I feel that this money may not be the uniform requirement of everyone. Sometimes, there are very poor patients for whom 500 rupees is insufficient. They may need more. You may also have a person coming from a very high-profile setting; for whom this money is not of much use because they can afford nutrition on their own. So, it is a diverse mix."*- District-level NTEP staff

Many beneficiaries were unaware that the NPY benefit was provided to meet their additional nutritional requirements during TB treatment. The health workers could not guide and support the beneficiaries regarding the use of the scheme amount due to various reasons, the inadequacy of the amount cited as the primary one. Due to the soaring food prices, there were few nutritional options to suggest to beneficiaries that would fit the monthly scheme amount. Hence, they suggested alternatives like regular provision of food kits or a combination of cash and kits on a case-to-case basis to enhance utilization.

"*I purchased fish, meat, eggs, and milk. The money was insufficient; I could barely manage, but 500 (rupees) is insufficient. For example, if we take half a litre of milk every day, then per month, it takes around 700–1500 rupees only for milk."*- NPY beneficiary

#### Delay and uncertainty in the credit of payments

The delay in crediting the amount and the uncertainty regarding the time of credit prevented beneficiaries from planning their allocation to procure healthy food. Many observed that they often received the scheme amount after treatment completion. The health workers also cited the delay in the credit of benefits as a barrier to adequately guiding the beneficiaries regarding its appropriate utilization. The dissatisfaction among the beneficiaries over the delay affected their receptiveness and responsiveness to the information offered by the health workers.

*"The program staff is not able to tell the beneficiaries, "you will definitely get the money by this day," right? We are not able to make that promise to the patient because there are a lot of processes which are independent of the program staff. So, if we can have such a system where our field staff can say confidently that by this date this much money will reach your bank account that would make a difference."*- District WHO Consultant

#### Banking-related issues

The beneficiaries do not always receive SMS notifications from banks or *Ni-kshay* on the credit of benefits regularly, preventing timely utilization. Illiteracy and difficulty comprehending the SMS that may not be in their mother tongue (native /regional language) also result in delayed or non-utilization of the benefit. When PwTB without bank accounts submit their family member's bank account details for the credit of the benefit, it hampered the beneficiary's direct handling of the benefit. In some instances, the banks had debited penalty from customers’ accounts (for not maintaining a minimum balance account or pending loan instalments) from the credited scheme benefit. In addition to this, not all beneficiaries are familiar and comfortable with technology and digital financial transaction which required them to travel to banks or Automated Teller Machines (ATM) to withdraw money to utilize them.

*“They (healthcare providers) did not say anything about what the money is for or when we will get it, just said that the government will give 500 rupees/month. I did not have any cash with me after the treatment. So when my daughter visited us, we asked her to check my bank account to withdraw whatever is remaining, and found that 3000 rupees have been credited. We did not know how it came or from where. We don’t usually go to the bank or check balance regularly. But then there was money in the account beyond our means, and we remembered that it may be from the government… we don’t remember getting any messages in phone”* -NPY beneficiary who completed treatment.

#### Transportation facilities and road connectivity

Where the terrain and weather are difficult and connectivity is poor, reaching banks or ATMs was both time-consuming and difficult for beneficiaries, which diminished their chances of utilising the benefits.

"*To open a bank account, they must travel and come to the taluk (sub-district headquarters) or town as they don't have a bank in their village. Also, the majority of the people here don't use ATMs; they have to go to the bank. To withdraw an amount of 500 or 1000, they should use their time to travel to the taluk and spend an additional amount for this travel."*- Field-level NTEP staff

## Discussion

This qualitative enquiry mapped the implementation processes of a direct benefit cash transfer scheme for PwTB in India, and explored the enablers and barriers to its effective implementation and utilisation. NPY is widely perceived as a beneficial initiative by all the stakeholders. Strong political commitment and innovations at the sub-national level were key enablers of effective implementation. However, the complex, multilevel implementation process, inadequate integration of the program activities with the overall public health system, and inequitable access to banking services challenged timely and optimal utilization of benefits.

Our findings indicate that the stakeholders and the beneficiaries perceived the scheme as beneficial. NPY benefited from political commitment and active involvement of the state in the implementation of the scheme. High political commitment along with powerful local leadership have been reported as essential factors determining the implementation and scale up of public health programs particularly in TB [24, 25]. The National Strategic Plan for TB elimination also emphasizes the role of local governments and local initiatives in TB elimination, along with the immediate need to integrate the general health system with the activities included in the TB program [26]. We found that health system initiatives at the national and state-level, such as digital application to track real time data or grievance redressal mechanism, worked in tandem with the scheme-specific efforts to enhance NPY implementation.

The process mapping highlighted that releasing monthly instalments involves creating, verifying and approving benefits in two digital platforms, dedicated human resources and several micro steps from field to district/state levels leading to considerable delays in credit of benefits. Delays are a key problem affecting the implementation of TB cash transfer programs, which erode the beneficiaries' trust in the programs [12, 14, 27–30]. They also deprive PwTB of support during a crucial early period of treatment when most unfavourable TB treatment outcomes occur [31, 32].

Opening bank accounts have been reported as a major hurdle faced by beneficiaries of direct-benefit cash transfer schemes in many contexts [14, 29]. Strategies like assigning a specific day in a week for bank account opening by the program staff have also been suggested [12].Our stakeholders also suggested formally engaging one or more public sector banks to facilitate the opening of new zero balance and maintenance accounts for every beneficiary newly enrolled into the scheme exclusively for the receipt of NPY benefits.

In many remote and hard-to-reach settings with poor physical connectivity, transportation costs impede the beneficiaries from accessing health services, reaching banks and utilizing the benefits. The support currently provided to cover the transportation costs for beneficiaries residing in hilly areas is insufficient. Reimbursement of the transport costs incurred in health care visits, opening bank accounts, and accessing the benefits may enhance utilization in such terrains [12].

Our study and previous studies reported challenges of poor internet connectivity, lack of adequate equipment such as computers/tablets, technical issues and software glitches related to *Ni-kshay* and PFMS portals [28, 29]. The field-level stakeholders observed that the *Ni-kshay* portal could be made more user-friendly if the common errors leading to non-validation of accounts and rejection of benefits, such as duplicate bank account numbers, unacceptable banks and so on, could be highlighted during data entry and not show up only when benefits are rejected. Lack of supportive supervision while navigating the portal and difficulty accessing technical support when required have also been highlighted as potential reasons. In many settings, the use of digital platforms for real-time data documentation, originally intended to enhance efficiency and accuracy, has added to the responsibilities of frontline health workers [33], taking away from the precious little time invested in patient engagement. Our stakeholders reported overburdening of the human resources and poor motivation posed major system-related challenges. Shortage of human resources, overburdening, hectic work schedules, and poor motivation of the program staff have been reported as challenges to TB-related cash transfer schemes in studies from several settings [14, 28–30]. The field staff's inadequate motivation and ability resulted in poor information transfer to the PwTB and inability to guide and support the beneficiaries to utilize the benefits effectively. This could spring from fundamental constraints in realistic task allocation, incentivizing and lack of monitoring. Supportive supervision, training, and regular and adequate financial and non-financial incentivisation, including recognition, opportunities for career development and appreciation from the community members, are crucial in motivating the health workers/volunteers [34–37].

The perceived inadequacy of the benefits given in NPY, which affected the appropriate utilization of benefits, has been reported in almost all the previous evaluations of NPY [28–30]. The CRESIPT project from Peru was guided by the notion that the incentive amount should be small enough to prevent its use as a perverse incentive but large enough to support the beneficiary and household and also decided by the intervention setting and proposed outcomes [12]. Multiple factors, including household size, structure and income, socio-institutional diversity across states in food, and infrastructure, inflation and price rise [38, 39], and market access of communities’ influence food-related expenditure and consumption patterns [40]. Cash transfer schemes targeting the poorest households in many settings like Ethiopia and Kenya regularly adjust the benefits in line with the inflation by monitoring the beneficiaries' purchasing power [41–43].

Our study indicated that many beneficiaries did not know how to utilize the benefits effectively to meet the additional nutritional requirements during treatment. Inadequate knowledge and stigma have been highlighted as barriers to accessing TB-related services in many settings [44]. It has been recommended that cash transfer schemes work with initiatives towards psychosocial support, education for individuals and participatory activities to tackle community stigma [12, 44]. There is a need to develop contextually relevant and culturally appropriate behavioural change communication strategies in local languages to nudge the beneficiaries towards utilization of benefits.

The fact that the scheme was not reaching the most deprived groups such as the homeless or the institutionalised, was a pressing concern. In addition to the existing challenges related to opening bank accounts, many of these sections do not have the provision to buy or cook food. Strong multi-sectoral collaborations, partnering with relevant civil society/non-governmental organizations, local self-governments and charitable institutions already working with such groups in specific contexts could be beneficial in identifying such PwTB, enrolling them into NPY and monitoring them.

Finally, TB is a disease of the households and not individuals, in terms of disease transmission, and its social and financial impact. In addition to TB-specific approaches like cash transfer schemes, TB sensitive approaches focusing on poverty reduction, income enhancement and improving food security in TB prevention must be considered.

Ours is the first evaluation of NPY implementation and utilization which included a nationally representative sample of respondents; both providers and beneficiaries. Our engagement with the national TB program since the design stage of this study not only encouraged active participation from the providers but will also promote their uptake of our recommendations to the program. It is possible that the providers refrained from sharing some of the pitfalls of NPY given that they were ‘insiders’. We have attempted to overcome that by assuring confidentiality and anonymity of data collection process. Though our study spanned thirty districts across nine states, it could have benefitted from a larger sample of beneficiaries to better capture a wider range of perceptions about the scheme.

## Conclusions

NPY is perceived as a beneficial initiative by both the providers and beneficiaries. Its implementation is enabled by strong political commitment at national and sub-national levels. The complex, multilevel implementation process of NPY, difficulties of facilitating unique bank accounts for all PwTB, overburdening of the program staff, inadequate integration of the program activities with the overall public health system, perceived inadequacy of the amount and inequitable access to banking services challenge timely disbursal of benefits and their optimal utilization.

We recommend that the program introduce greater operational simplicity in NPY implementation by reducing the number of instalments and levels of verification to ensure timely receipt of benefit.

Mainstreaming NTEP activities with the public health system could lead to effective task allocation and sharing among NTEP staff allowing more time for engagement with PwTB.

The benefit amount could be equitably revised, acknowledging the extent of social and financial vulnerability of the households and the members' purchasing power by inflation.

Strengthening partnerships with civil society/non-governmental organizations, local self-governments and charitable institutions to design and deliver care for nutritional support of vulnerable populations with PwTB.

### Supplementary Information

Supplementary Material 1.

Supplementary Material 2.

Supplementary Material 3.

Supplementary Material 4.

## Data Availability

The transcripts generated and/or analysed during the current study are not publicly available due [to protect the participants’ anonymity and data confidentiality] but [fully anonymised codebook] can be made available from the corresponding author on reasonable request.

## Abbreviations

ASHA: Accredited Social Health Activist
ATM: Automated Teller Machine
CTD: Central Tuberculosis Division
DBT: Direct Benefit Transfer
DTO: District Tuberculosis Officer
NGO: Non-Governmental Organization
NPY: Ni-kshay Poshan Yojana
NTEP: National Tuberculosis Elimination Programme
PFMS: Public Finance Management System
PPSA: Patient-Provider Support Agency
PwTB: Persons with tuberculosis
SMS: Short message service
STO: State Tuberculosis Officer
STS: Senior Treatment Supervisor
TU: Tuberculosis Unit
WHO: World Health Organization
