# Supplementary material for: Need for operational simplicity and timely disbursal of benefits—a qualitative exploration of the implementation of a direct benefit transfer scheme for persons with tuberculosis in India
Source: Infect Dis Poverty. 2024 May 23;13:36. doi: 10.1186/s40249-024-01206-3 (PMC11112885; doi:10.1186/s40249-024-01206-3)
Supplement: Supplementary file 2 — Supplementary Material 2. [file 40249_2024_1206_MOESM2_ESM.pdf]

In Depth Interview Guide

(Field health workers/volunteers from the public health system and non-governmental organizations).

Name of the participant:
Designation:
Date of Interview:
Interview start / end time:
Name of the Interviewer:

After a brief introduction to the participant regarding the purpose of the interview, the interviewer will take informed written consent for the interview. Written informed consent will also be requested for audio recording.

1. Could you briefly describe your professional trajectory and about your current role and responsibilities particularly related to NPY implementation?
2. We would like to know your opinions about the Ni-kshay Poshan Yojana. – the cash incentive scheme via direct benefit transfer for TB patients. As per your opinion, what are the positives about this scheme?
3. Could you briefly take us through the key processes involved in implementing NPY?
4. What do you think of the performance NPY implementation over the last five years? Could you explain the reasons for this assessment?
5. How did the already existing mechanism in health system support the implementation of NPY? What additional factors were/are added for its implementation? [Human resources, infrastructure, funds to achieve the programmatic goals]
6. Could you briefly take us through your routine tasks under/related National TB Elimination Program, particularly related to NPY?
7. What factors act as facilitators in implementing this scheme? [Probe- programmatic, patient level]
8. What specific factors support/enable your work involved in NPY?
9. Based on your experience, have you found any barriers that hinder the effective implementation of this scheme? [Probe- programmatic, patient level]
10. What specific factors hinder your work involved in NPY?
11. What could be the solutions for these barriers as per your opinion? [Probe- programmatic, patient level]
12. What do you think about the promptness in delivery of the benefits? Have you observed any delay in payments? If yes, what are the reasons? [Probe-programmatic, patient] What are the possible solutions?
13. We have come to realize that unique beneficiary bank accounts are required. How do you manage to ensure this for all the beneficiaries/ [probe further on people who are vulnerable or marginalized]
14. Could you briefly explain the monitoring and supervision of the scheme implementation? [How often are review meetings conducted? Who all are the attendees of the meetings? What are the discussion points usually?]
15. We would like to know more about the grievance redressal mechanisms [What are the mechanisms to address the complaints from the field level implementers and beneficiaries? What kind of complaints do you usually raise? How promptly are the complaints addressed? What kind of challenges do you face in this regard]
16. How would you characterize your experience with the Nikshay portal? [Training received, ease, challenges in navigating the portal]

17. How far has the program been integrate with the routine health system activities? [task allocation of human resources, coordination with the other health system players]
18. Could you let us know about any specific state level or regional or local initiatives to implement NPY, which exist in your state/district? [Players in such innovations - local bodies/other health system players/bank officials]
19. Additional Remarks

Interviewer will complete the interview by acknowledging the participant for participation. They will also share the summary of the notes taken and take confirmation from the participant.
