# Supplementary material for: Need for operational simplicity and timely disbursal of benefits—a qualitative exploration of the implementation of a direct benefit transfer scheme for persons with tuberculosis in India
Source: Infect Dis Poverty. 2024 May 23;13:36. doi: 10.1186/s40249-024-01206-3 (PMC11112885; doi:10.1186/s40249-024-01206-3)
Supplement: Supplementary file 3 — Supplementary Material 3. [file 40249_2024_1206_MOESM3_ESM.pdf]

In Depth Interview Guide

(Patients notified with TB enrolled under NPY).

Name of the participant:
Date of Interview:
Interview start / end time:
Name of the Interviewer:

After a brief introduction to the participant regarding the purpose of the interview, the interviewer will take informed written consent for the interview. Written informed consent will also be requested for audio recording.

1. Could you briefly describe your personal and family situation? [Personal background, education, occupation, family members, means of livelihood, income and so on]
2. We would like to know a little bit about the circumstances of your diagnosis and treatment? What is the present status of treatment? [Appreciating the patient for completion of treatment].
3. Have you heard of any cash incentive scheme for TB patients? If yes, can you tell me about details of it?
4. How did you receive the information about cash incentives? What were the instructions given to you?
5. Have you received any cash benefit during your course of treatment? [If they respond yes, then probe the following]

    a. Could you share with us the details? [Probe- Number of installments, Frequency of installments, Timing of installments]
    b. Could you briefly describe the experience of receiving these benefits
        i. How easy/difficult was the process of receiving the benefits? What kind of problem(s) have you faced, if any? (Probe: number of visits to the facility, bank, home visits from the NTEP staff, process of enrollment and so on)?
        ii. As per your knowledge, what were the reason(s) for the same?
        iii. What do you think of the cash incentive scheme? [Utility, purpose of the scheme, what did they use it for specifically, amount, sufficiency, issues in accessing the benefits, services etc.]
        iv. When you faced these challenges how did you deal with it? [who did you approach, what kind of response did you get]

6. What could be the reasons that you have not yet received the money? [for those patients who have not heard of the scheme/received the money yet]

7. Additional remarks, if any.

Interviewer will complete the interview by acknowledging the participant for participation. They will also share the summary of the notes taken and take confirmation from the participant.
